# Malpractice

**Published:** 1889-12

**Authors:** Lucius Weinschenk

**Affiliations:** Attorney-at-Law, Chicago


Contributed exclusively to Daniel's Texas Medical Journal.

### MALPRACTICE.

BY LUCIUS WEINSCHENK, ESQ., ATTORNEY-AT-LAW, CHICAGO.

Read Before the Chicago Medico-Legal Society, October 5, 1889, President E. J. Doering, in the chair.

OF ALL the learned professions, that of the physician is of the greatest concern to the majority of mankind. It deals directly with our health, the proper maintenance of which lies at the very foundation of a useful career in life, without which happiness is unknown. The priest or pastor is concerned only with such persons as are of a spiritual turn of mind, and who profess religion, prompted either by sentiment or conviction. These votaries of conscience form but a fragment of the race. The lawyer sacredly guards the interests of those who either from accident or misfortune seek his aid. He is the protector of their person and their property, the guardian of their liberties. Here too, those dependent upon this profession, form but an increment of society. But the doctor is intrusted with the very existence of life and the health and happiness of those who are most dear and near to us. The rich and the poor, the proud and the humble, the old and the young, all are alike dependent upon the physician from the very moment in which they are ushered into existence until such time as death seals the lips, and the soul wearied with the hardships of terrestrial servitude seeks a more glorious existence. Of the great triumvirate of professions, therefore, law, theology and physic, that of the physician may be regarded as pre-eminently the noblest and most useful of human pursuits, having for its aim the temporal concern of man. It is the prime factor in the production of happiness and prosperity. By reason of the very law of nature by which man was deprived of eternal life and incurred the liability to corporeal disease—the healing art is co-eval with man himself; necessity alone prompted its inception, self preservation urged its study, reason and experience promoted it, until to-day it stands as a living monument to testify to the great skill of those who devoted years to its study and gave their best efforts for the benefit of suffering humanity.

To no class of persons is the esteem and veneration of mankind so justly due as to him who devotes his energy and life work to the amelioration of his fellow man, and whose aim and purpose is to so help his neighbor that he may properly perform his alloted task in life. While the very calling of a professional life is generally of itself a guarantee that those who follow it are men of honor, integrity and principle, it is, nevertheless, a fact which none will dispute, that in every profession there are those whose nature properly calls them to other careers more in keeping with their habits of life, and who would honor their vocation by their absence; this is true none the less in the practice of medicine than in any other walks of professional life. That the majority often suffers by reason of the vices of the few is so axiomatic that it hardly needs repetition; this fact, together with the selfish and wicked designs or evil intentions of many, have often brought upright and well meaning men who have tried to do their duty honestly and conscientiously, into disrepute, and it has been a matter of no rare occurrence that a doctor who has treated his patients conscientiously, giving them the benefit of his best thoughts and efforts, and sparing neither time nor patience in their behalf, is often villified and traduced by reason of alleged injuries which exist only in the hallucinations of those who either mistakenly, or from malice aforethought, consider themselves wronged.

Increasing civilization has produced a singular state of facts in so far that man's moral training has failed to keep pace with his intellectual advancement. This is largely due perhaps to the materialistic tendency of modern days as well as the desire of opulence, which is the ambition of the masses, and to attain which, intellectuality is often strained in every direction, to the exclusion if necessary, of morality.

The best guage and standard of the greed of mankind is the law reports which contain accounts of suits for damages, brought for injuries supposed or real; to be sure a great many such are founded upon a meritorious state of facts; but on the other hand, it happens that every conceivable artifice is often resorted to, deceptions of all kinds practiced, and legal technicalities of every nature invoked, to cause such as are accused of wrong doing, either through negligence or design, to pay the penalty of their acts. In this respect the dignity of the physician has not shielded him from attack by scheming, conniving and malevolent persons who seek to gain pecuniary benefit for imaginary injuries, born of the greed and malicious motives of dishonorable men. Not but that there are cases honestly brought and in which physicians are oftentimes rightly made to pay the penalty of negligence, whether wilful or accidental, but the great majority of such as are recorded in our reports of courts of last resort are found to be chimerical in their inception, fraudulent in their design, and brought for evil motives, and thus they meet with such end as they justly merit; such and kindred cases, be they meritorious or otherwise, have been of so frequent occurrence during the present century that they have formed, as it were, a separate branch of the law, known as medical jurisprudence, and many eminent writers have spent years of their life in the study of this branch of the profession and written learned treatises upon this subject.

There is one class of cases which, while it is not properly classed in the category of medical jurisprudence, the physician whose practice lies in large cities, and who is called upon to deal with all elements of society, is most often concerned with; it is the case of malpractice. To briefly consider such of its features as are of importance to the medical practitioner, is the purpose of this paper.

All actions brought against physicians by reason of alleged negligence in the treatment of patient occasioned by malpractice or other dereliction of duty, must in this State, by virtue of our statute of limitation, be brought within two years after the alleged cause of injury took place. In all such cases the plaintiff in his declaration, the instrument in which he discloses his cause of action, not only alleges the elements of carelessness and negligence, but also, generally, the absence of knowledge or skill.

In medics as in law, the practitioner is not responsible for the errors of an elightened judgment. At the same time, he is not at liberty to interpose his judgment contrary to that which is settled. How unjust it would be to permit a physician to experiment with his patient beyond the settled rules of the law. What are the settled rules of the law? None but the most general tests can be stated as comprehended in modern statutes. The great variance between the medical theories that find adherents in this age, makes it impossible to assert as a proposition of law that any particular system affords an exclusive test of skill. The character of the disease, the habits and tendencies of the patient, both, determine the amount of skill required to satisfy the canons of the law. A physician about to administer an anæsthetic, is bound to inform himself as to the condition of the patient's heart and other organs, which if diseased, would warn a prudent physician against the administration of that beneficent agent.

It is a matter of great importance to physicians to learn just what laws our courts of last resort have laid down as to the requisite degree of skill which it is necessary for a physician to possess, to shield him from such actions, provided, his treatment in all cases, is prescribed in accordance with the recognized rules of practice in each particular case. The supreme court of Illinois, in the case of Hallem vs. Means, reported in the 82 Ill. R. page 379, have laid down the following as a proposition of law: "While perhaps persons to hold themselves out to the public as physicians and surgeons, would not be required to possess the higest degree of skill which the most learned might acquire in the profession, yet they are bound to possess, and in their practice to exercise the degree of skill which is ordinarily possessed by physicians in practice, and where an injury results from a want of ordinary skill, or from a failure to exercise proper diligence and caution in the treatment of a case, the physician must be held responsible." This is the well settled law, not only in Illinois, but in all the states of the union, and the courts of last resort have universally followed it where malpractice cases have come before them for final decision.

The rule again is well laid down by another court in the following words: "Whoever undertakes to practice physic or surgery holds out to the public that he possesses a competent degree of medical skill, according to the general character of the medical science in the section of the country in whice he lives. The degree of professional talent which may be expected, will depend much upon the patronage and encouragement by which it may be fostered and elicited. In large and opulent towns and cities where physicians and surgeons find extensive employment and ample compensation, competition is invited, and the candidates for public favor in medical attainments are stimulated by the most powerful motives in their endeavors to attain professional eminence, and are at the same time aided by many facilities not to be found in more secluded and less favored situations. The highest degree of skill therefore is not to be expected in small towns where there is little competition and fewer motives for exertion, for the comparative want of patronage, and the limited opportunities afforded for professional improvement." From this decision it would seem that the test of proper medical treatment in each case is the skill possessed by the practitioners in the community in which the defendant in a malpractice case may reside.

In medical jurisprudence as in other branches of the science of law, the element of contributory negligence forms a conspicuous part in determining the relative rights of the parties. A patient cannot recover for injuries consequent upon negligent treatment by his physician, if his own negligence contributes to the result. If it be impossible to separate the injury occasioned by the relative neglect of each, the patient cannot recover. Again, it is held that if a person while under treatment injures himself of his own carelessness, and the physician afterwards treats him unskillfully, the patient may recover for any distinct injury resulting from such unskillfulness. Then too, the patient is required to adopt and follow out all reasonable directions and requirements of the physician relating to the treatment of the disease or the injury, and if he fails so to do, and injurious consequences, affecting the disease or injury, result from his failure, he cannot recover of the physician or surgeon by alleging a want of skillfulness on the part of such physician or surgeon. As a general rule, malpractice suits are not looked upon with favor by our courts, and judges, and the plaintiff will be held to observe the strict rules of evidence applicable to the case at bar. These rules comprehend the burden of proof in which the plaintiff is compelled first to establish some positive act of negligence or misconduct.

A principle which pervades the law but finds its especial application to the matter under consideration is, "That no man can take advantage of his own wrong or charge his misfortune to the account of another."

Perhaps the greatest things physicians have to fear in the trial of malpractice cases in nisi prius courts, is the fact that our juries, as a rule, are largely composed of men, who while they are actuated by the best motives and by a desire to do what is right and proper in each particular case, are, nevertheless, through ignorance of what may properly be taken into account in forming their verdict in such cases, led astray, so that in the majority of cases, erroneous opinions and impressions are formed. The jury is often swayed by reason of sympathy which they may feel for a person who has sustained an injury, and various other devices are brought to bear to influence them in the rendition of their verdict. Not being versed in the rules of the law, persons sitting upon juries are apt to decide rather from what, excited by their passion and sympathy they may consider right, than solely by the weight of the evidence and the law in each particular case. It is true, of course, that each jury is instructed as to how verdicts are to be arrived at, but it also is the universal experience of bench and bar, that but little atttention is paid to such instructions. From the law as above laid down, it follows that if a physician will at all times use the highest degree of care and skill of which he is capable, and follow out the regular and recognized methods of procedure and practice in each particular case, no action of malpractice can be successfully maintained against him, and in the event that a verdict is recovered against him, upon which no new trial is granted, and such have been many, in the annals of our jurisprudence, courts of appeal have universally interfered and set such verdicts aside.

Occasionally there arise in the decisions of higher courts opinions that are at once interesting, original and unique. A very sensible one is to be found in the case of DeMay vs. Roberts, 9 N. W. Rep., p. 146. In this case a physician takes an unprofessional unmarried man with him to attend a case of confinement where there was no emergency requiring the latter's presence. The evidence recites that the physician told the patient's husband that he had brought a friend with him to help carry his things, and he was accordingly admitted. The patient, on afterward discovering the facts, sued both in damages. Judgment was rendered in the lower court in favor of plaintiff, and the decision was affirmed by the supreme court of Michigan. The judges of the latter court, in rendering their decision, said: "It would be shocking to our sense of right, justice and propriety to doubt that for such an act the law would afford an ample remedy. To the plaintiff the occasion was a sacred one, and no one had a right to intrude, unless invited, or because of some real or pressing necessity, which, it is not pretended, existed in this case. The plaintiff had a legal right to the privacy of her apartments at such a time, and the law secures to her the right by requiring others to observe it and to abstain from its violation. The fact that at the time she consented to the presence of a stranger, supposing him to be a physician, does not preclude her from maintaining an action and recovering damages upon afterward ascertaining his true character. In obtaining admission at such a time and under such circumstances, without fully disclosing his true character, both parties are guilty of deceit, and the wrong thus done entitles the injured party to recover damages sustained from shame and mortification, upon discovering the true character of the defendants."

There is, perhaps, no situation which is so delicate to a physician as when placed upon the stand as a witnesss in a cause in which testimony may be required of him. The property, reputation, liberty, or even life itself, of any member of society, may be vindicated or protected, or blasted and destroyed by the fidelity or recklessness of the medical witness. As a matter of common law, there is no privilege of communication between physician and patient, such as exists at law between attorney and client. In the latter case, all communications made by a client to his legal adviser, concerning a matter at issue, are known as privileged communications, and the lawyer cannot be compelled to disclose them upon the witness stand. Such privliege did not exist at common law as stated, between doctor and patient, nor does it to-day exist in our own State. A great many States of the Union have, however, incorporated into their code a statute similar to one in vogue in New York State that runs as follows: "A person duly authorized to practice physic or surgery shall not be privileged to disclose any information which he acquired in attending a patient in a professional capacity, and which was necessary for him to act in that capacity." Under this law it was held that information not only means communications received from the lips of the patient himself, from the statement of others who surround him, or from observations of his appearance and symptoms. This statute creates a privilege merely in favor of the patient, hence, after the death of the patient there is no person entitled to assert it, so that, for instance, a physician may be called upon to testify as to the mental capacity of a deceased patient in the probating of a will and similar cases. A statutory law substantially the same as exists in the State of New York, is found upon the statute books in the following States of the Union, namely: Missouri, Wisconsin, Iowa, Indiana, Arkansas and California. The law of Illinois, however, upon this subject remains the same as it was laid down in the celebrated Duchess of Kingston cases, in which it was held that "in a court of justice medical men are bound to disclose such secrets as may have been imparted to them by their patients, when required to do so." In that case Lord Mansfield says: "If a medical man voluntarily imparted these secrets, to be sure, he would be guilty of a breach of honor and a want of discretion, but to give that information, which by the law of the land he is bound to do, will never be imputed to him as any indiscretion whatever. With respect to his knowledge of criminal acts, when not called upon to testify with reference thereto, the universal professional code of ethics simply demands that a physician is not to play the part of spy or informer, and no matter how strong may be his moral convictions, he should nevertheless consider himself under an equivocal bond of secrecy, if, solely through his professional knowledge and dealings he may have learned that his patient is an escaped convict or criminal, no matter how heinous may have been his crime. The criminal has a right to medical services in sickness, and the duty of the phycician in such cases relates exclusively to the patient. As it is the object of this organization to foster the interests of society from a medical standpoint, allow me to suggest that appropriate steps be taken for the enactment of some such statute relative to the protection of professional secrets, as we have seen exists in other States. Such statute would not only be of great benefit to the community at large, but would save the physician much time, trouble and annoyance, which he is now put to in attendance upon court, oftentimes for several successive days, for the purpose of giving some testimony, which, peihaps, is a matter of but slight importance, and by reason of which other and necessary business is allower to suffer. Were such a law upon onr statute books, the physician would very rarely be called into court, except on such occasions as might not be covered by the proposed enactment.

In closing this paper, our regret is that the data at hand and the ability to prepare it for your consideration, have both been so exceedingly deficient, but, unlike the unfortunate Jew, who journeyed to Jericho, we are intuitively conscious that it has fallen into the hands of indulgent friends.

DISCUSSION.

DR. J. H. ETHERIDGE:—Mr. President: Very many malpractice suits start from the medical profession. In fact, it is not an uncommon thing for a lawyer with his client to go around to doctors to get opinions on the treatment of such and such a case, and oftentimes a case of malpractice is started on the opinion of a doctor. In communities where there is a low state of esprit du corps, there are more malpractice suits than where this is high. Dr. Ray, of Louisville, recently told me that they never had malpractice suits in Louisville; they never had criticism of treatment, and the result was that when patients who had a grievance, went around to get opinions of other doctors, they had no opinion to give. Which reminds me of a distinguished physician in our midst, who was called upon by a lawyer and a young boy. The moment they came in the door he made up his mind that it was a lawyer and client. The lawyer got the boy in front of the doctor, and pulled up his sleeve; the boy had a turned down wrist, and the lawyer said, "Doctor, what do you think of that as the result of a fracture?" The doctor said, "Sir, I think that is a—good result." It happened the next day when he was lecturing on Colles's fracture, he espied that lawyer on a top seat, and malpractice suits got a thorough ventilation. It is so very common among a certain class of physicians to give adverse opinions on the work of other doctors that it is no wonder we have malpractice suits. Until physicians have that sense of honor which will make them refrain from giving invidious opinions on the work of other physicians, malpractice suits will thrive. Occasionally there is good cause for malpractice suits, and in that case I think the doctor had better consult a lawyer very quickly. Dr. Brainard told years ago the following: "A celebrated surgeon living in Rochester, N. Y., wrote to many physicians and wanted to get an opinion on a certain case. A few months before, a man had come to him who was so troubled with the sexual instinct that he was beyond all reason, and he had come to have his testicles removed. The doctor was prevailed upon to remove the testicles, but a few months afterwards the man wanted to get married, and he thought he would have a good suit against this physician. The physician wrote to Dr. Brainard, who advised him to take the man in the back room and settle with him, as no justifiable cause existed for the removal of these organs."

When a malpractice suit is sprung upon a physician it means business, and it should be treated as such, and the doctor who looks upon it as trivial, and treats it in a dilatory sort of a way is the man who is going to be worsted. There are several good points that should be considered. One is to engage the services of a good lawyer. It is unnecessary to say that when people are sick they should get a good physician, and when we are sick in a legal way we also should get a good legal physician. Another thing physicians should do, is this: after securing a lawyer they should go to work and instruct him well in regard to the case. Where there are fractures and ligations, it would be a good thing to get a subject and dissect it, and instruct the lawyer so that he can go to work and do the thing that has been successfully done in these suits, namely, smash the expert, squeeze him to death, on his anatomy. The doctor who has taken the pains to so instruct his lawyer will enable him to go to work on his examination of the witness for the plaintiff, and make him sick on the witness stand. That can be done; of course it takes enterprise, but he can do it. Another thing that is of advantage to the physician, is to secure all similar cases in print; judge and jury want precedents and lawyers and plaintiffs don't, and whenever we can gather them up in the literature of our own, or the legal profession, it is better to do it and pile up precedent on precedent, one after the other, and in that way mass the evidence so overwhelmingly on the side of the physician that there is little left for the plaintiff to work upon. Another thing: It is important to secure good, competent witnesses who will testify in the right sort of way, who understand the spirit of the case, and who do not want to answer questions where they run pretty close to the nerve. A man of celebrity may not be too well educated, and when we have a witness of that sort we want to educate him and feel sure that he can answer all the questions. Another qualification of a good witness is truthfulness. Lying never helps any case. Truth is like silver, the more you burnish it the more it shines. The man who has to bolster up his case by lying is in an awfully ticklish position. Another point of interest to the physician is the comprehending of his own case in all its bearing, and in every way. The physician who goes before the court relying upon the wisdom and skill and testimony of anybody else, without thoroughly comprehending his case, is in a great many instances going to be beaten, and the physician who comprehends his case in all its bearings is going to win. I must confess, I have never seen a malpractice suit in which the physicians in this state have been to blame. Occasionally it looks as though a great deal of criticism might be made, but I think when we get the statement of the physician and all the facts in the case and all its bearings, there is always a little justification. When this society was organized the opinions of physicians and of several lawyers were obtained in writing and published in a pamphlet. I was particularly impressed with the opinion of one firm of lawyers; they said that more than nine-tenths of the malpractice suits were blackmailing suits. That is in black and white, and when lawyers of experience express such an opinion it seems to me that physicians have right on their side.

DR. J. R. BRANDT: Mr. President: A few years ago there came to my office a man who represented himself as an insurance agent, and brought with him a gentleman who desired to be examined. I said to him that I was not an examiner for that or any other company. He said, it made no difference in that matter, he only desired a very careful examination of the gentleman. I examined him and asked a few questions, not very many, but he was very particular that I should give a thorough examination, which I did. About an hour after this examination had taken place, I was summoned to appear in court. It seems that he had brought this man to me for the purpose of securing my opinion in regard to certain injuries that he had received in a railroad accident. This was not a blackmailing suit, nor a suit for malpractice, but we can see very readily how an examination and opinion might be secured by a similar procedure.

MR. LYNN HELM, Attorney-at-Law: I was very much impressed with what Dr. Etheridge said about instructing counsel, in any suit for malpractice, that might be brought against a physician, as to all things that will affect the case in point. I once heard a very distinguished lawyer, one who, before his death, tried perhaps more cases than any lawyer at this bar, and who was an authority in Northern Illinois and Iowa, say that he could try any case and upset any physician on the witness stand. He said that when he examined a physician on any particular thing, he always studied up the particular disease or accident, or whatever it might be that had happened to the one injured, and, he said, he never went rambling around the human body, but confined himself to that particular part, and the chances were that every physician who came on the stand, would come with his head full of the anatomy of the entire human body, and would not think of that particular thing when he came to be a witness, and so he would be able to entrap him. He said he could do this in almost every case. He instanced one case where a young physician of this city was sued for some surgical operation. Dr. Gunn was on the witness stand, and the examination was confined so closely to one point that the doctor, although testifying in favor of the defendant, said that the operation was unskillfully performed. It was all due to the manner of examination by counsel,—because of his confining his examination to that one particular point. He said, also, that sometimes a physician would be prepared for such an emergency and then he had to ramble around the human body generally, to see if he could entrap him.

There is one thing in regard to this subject of malpractice and blackmail that I have thought of especially, and that is, physicians need more than anything else strong backbone; they must not be afraid of malpractice suits, and thereby give up anything of their profession. Malpractice suits are seldom successful, and if successful, it is only because of the grossest neglect on the part of the physician. The physician and the lawyer are on a par, they are only required to use what is termed ordinary skill and ordinary knowledge. Of course, if a man guarantees to do the impossible, he is not to be held accountable for not doing it. If I agree to win a lawsuit and thereupon shall depend my pay, and I do not win it, I cannot recover anything, and if a physician agrees to cure a man and does not do it, he cannot recover his pay. But if a physician, in the ordinary course of practice, uses ordinary care and skill, he will not be subject to successful malpractice suits. This was illustrated in the quotation in Mr. Weinscheck's paper, taken from McClellan on Malpractice, a very interesting work, which lays down the entire general principle of what is required of the physician in his practice, and what would be malpractice in case he did not live up to its requirements. I do not think, however, that there is very much danger from these blackmail suits. Of course, any man can bring a suit; that is the privilege of any one, rich or poor, who can get a lawyer, and you cannot prevent them. You must defend these suits, and you should make it your business to attend to it, and the chances are all in your favor if you do attend to it. I do not know of any successful suits brought here in the last six or eight years against a physician because of malpractice. I think, physicians should go ahead in their work and practice their profession without thinking of malpractice suits, using the highest possible skill, and using their judgment without fear. A physician who would slight his profession for fear of a malpractice suit, is like the young woman who was a good housewife but not a good home maker, who kept the windows so clean that she was afraid to have you look out of them for fear they might be stained with the color of your eyes. And so I think, if you are too careful you will not be successful.

